# Fetal Presentation of Walker–Warburg Syndrome With a Novel POMT1 Splice‐Altering Variant: Antenatal Imaging, Postmortem MRI, Autopsy, and Molecular Correlation

**DOI:** 10.1002/ccr3.72716

**Published:** 2026-05-17

**Authors:** Jing Zhang, Pin Wang, Gan Tian, Yu Hu, Xin Zhang, Xiang Huang, Xiaoqiang Zhou, Peng Huang, Fengying Chen

**Affiliations:** ^1^ Department of Radiology, the Affiliated Foshan Women and Children Hospital Guangdong Medical University Foshan Guangdong China; ^2^ Department of Ultrasound, the Affiliated Foshan Women and Children Hospital Guangdong Medical University Foshan Guangdong China; ^3^ Department of Prenatal Diagnosis Center, the Affiliated Foshan Women and Children Hospital Guangdong Medical University Foshan Guangdong China; ^4^ Foshan Institute of Fetal Medicine, the Affiliated Foshan Women and Children Hospital Guangdong Medical University Foshan Guangdong China

**Keywords:** brainstem, MRI, POMT1 splice‐altering variant, prenatal diagnosis, Walker–Warburg syndrome, ‘Z’‐shaped

## Abstract

Walker–Warburg syndrome (WWS) is a fatal autosomal recessive disorder characterized by brain and eye malformations, and prenatal diagnosis relies heavily on neuroimaging findings to guide targeted genetic screening. Here, we describe a distinctive second‐trimester fetal imaging pattern observed in two siblings. In vivo and postmortem fetal magnetic resonance imaging demonstrated hydrocephalus, fused ventricles, hypoplastic cerebellar hemispheres, a small supraoccipital meningoencephalocele, and a recurrent hypoplastic ‘Z’‐shaped brainstem configuration. Whole‐exome sequencing identified a homozygous deletion in POMT1 (c.123‐11_123‐5del), confirmed by Sanger sequencing, and RNA sequencing suggested reduced expression of exon 3. Recognition of a ‘Z’‐shaped brainstem on fetal MRI should raise strong suspicion for WWS associated with POMT1 mutations, prompting integrated pathological assessment and genetic testing, particularly when there is a positive family history.

## Key Clinical Message

A hypoplastic ‘Z’‐shaped brainstem with hydrocephalus and cerebellar hypoplasia on fetal MRI should prompt suspicion of Walker–Warburg syndrome, especially with recurrent affected pregnancies. Integrating postmortem MRI, autopsy, WES, and RNA analysis can clarify POMT1‐related disease and guide targeted recurrence counseling.

## 1 Introduction

Walker–Warburg syndrome (WWS) (OMIM*613155) is the most severe phenotype within the dystroglycanopathy spectrum and is closely related to muscle–eye–brain disease (MEB). It is characterized by brain and eye malformations and is believed to result from mutations in glycosylation enzymes [1]. WWS is inherited in an autosomal recessive pattern, with POMT1 mutations accounting for a significant proportion of cases [2, 3]. As a severe and often fatal congenital disorder, WWS highlights the importance of prenatal diagnosis for prognostication, management, and parental counseling [4]. Currently, the prenatal diagnosis of WWS primarily relies on neuroimaging findings to guide targeted genetic screening. Although several antenatal ultrasound (US) studies have examined cerebral dysgenesis in WWS, routine US imaging is often insensitive and nonspecific for meeting diagnostic criteria [5, 6].

This study reports specific magnetic resonance imaging (MRI) neuroimaging findings of WWS during the fetal period. To our knowledge, this report provides a detailed fetal‐period correlation of in vivo MRI, postmortem MRI, autopsy, and molecular findings in Walker–Warburg syndrome associated with a homozygous POMT1 splice‐altering variant. The main novelty lies in the recurrent recognition of a hypoplastic ‘Z’‐shaped brainstem in two affected siblings during the early second trimester, which may serve as an imaging clue to prompt targeted molecular testing and recurrence counseling.

## 2 Materials and Methods

### 2.1 Case Report

**FIGURE 1:**
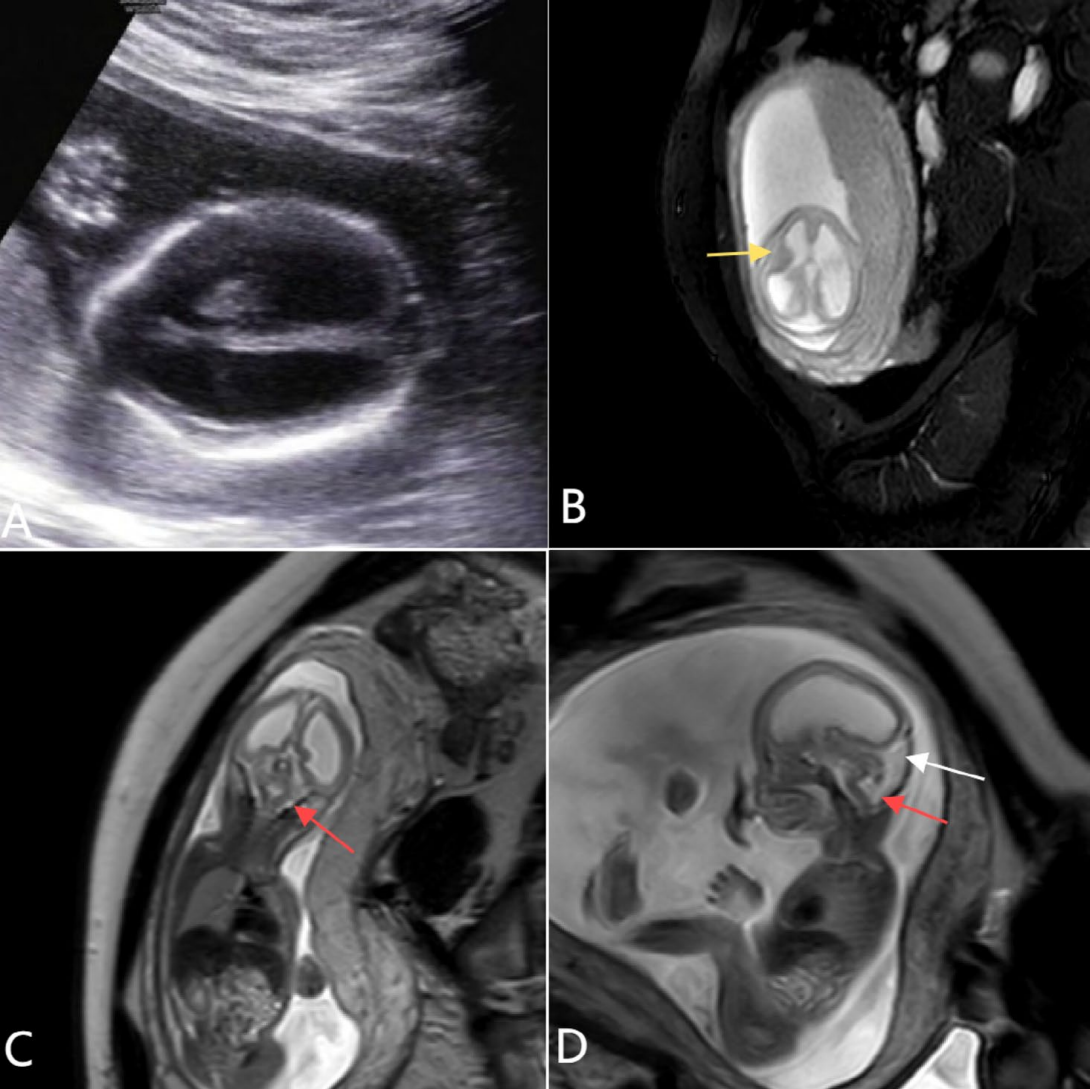
Images from Case 1. (A) US imaging at 19 weeks and 1 day shows severe triventricular hydrocephalus. Supratentorial sonographic images display an outer agyric echogenic band on the brain surface, along with a complete absence of the pericerebral space. (B–D) In vivo MRI at 22 weeks reveals axial T2—weighted imaging, which shows agyria and the absence of the pericerebral space, leading to interhemispheric pseudofusion. Severe ventriculomegaly is observed, along with periventricular cortical nodular heterotopias, indicating a neuronal migration disorder (yellow arrow). The sonographic midsagittal image shows a ‘Z’—shaped appearance of the brainstem (red arrows), agenesis of the corpus callosum, and a small vermis (white arrows).

The family history of the patient dates back to 3 years, when a 24‐year‐old primigravid woman was referred to our institution at 19 weeks and 1 day of gestation due to abnormalities detected during a routine US examination. The US revealed an abnormally enlarged posterior cranial fossa (Figure 1A). The parents appeared normal and were unrelated by consanguinity. At 19 weeks and 4 days of gestation, the woman underwent fetal MRI using a 3‐Tesla unit (Philips, Ingenia 3.0 T) equipped with a multi‐channel phased‐array coil. During the examination, a supine position with the feet first was used. The fetal MRI protocol included axial and sagittal T1‐weighted imaging, axial, sagittal, and coronal T2‐weighted imaging, axial fluid‐attenuated inversion recovery, and diffusion‐weighted imaging. The fetal MRI revealed severe brain dysplasia: the corpus callosum was absent, with significant enlargement of the bilateral lateral ventricles. The cerebellar vermis was not clearly visible, and the cerebellar hemispheres were significantly shrunk. Other findings included cobblestone lissencephaly, collicular fusion, hydrocephalus with fused ventricles, hypoplastic cerebellar hemispheres and vermis, and a pontomesencephalic kink (Figure 1B–D). Amniocentesis revealed a normal karyotype. A pregnancy was terminated upon parents' request; however, genetic testing and autopsy were declined due to cost concerns.

**FIGURE 2:**
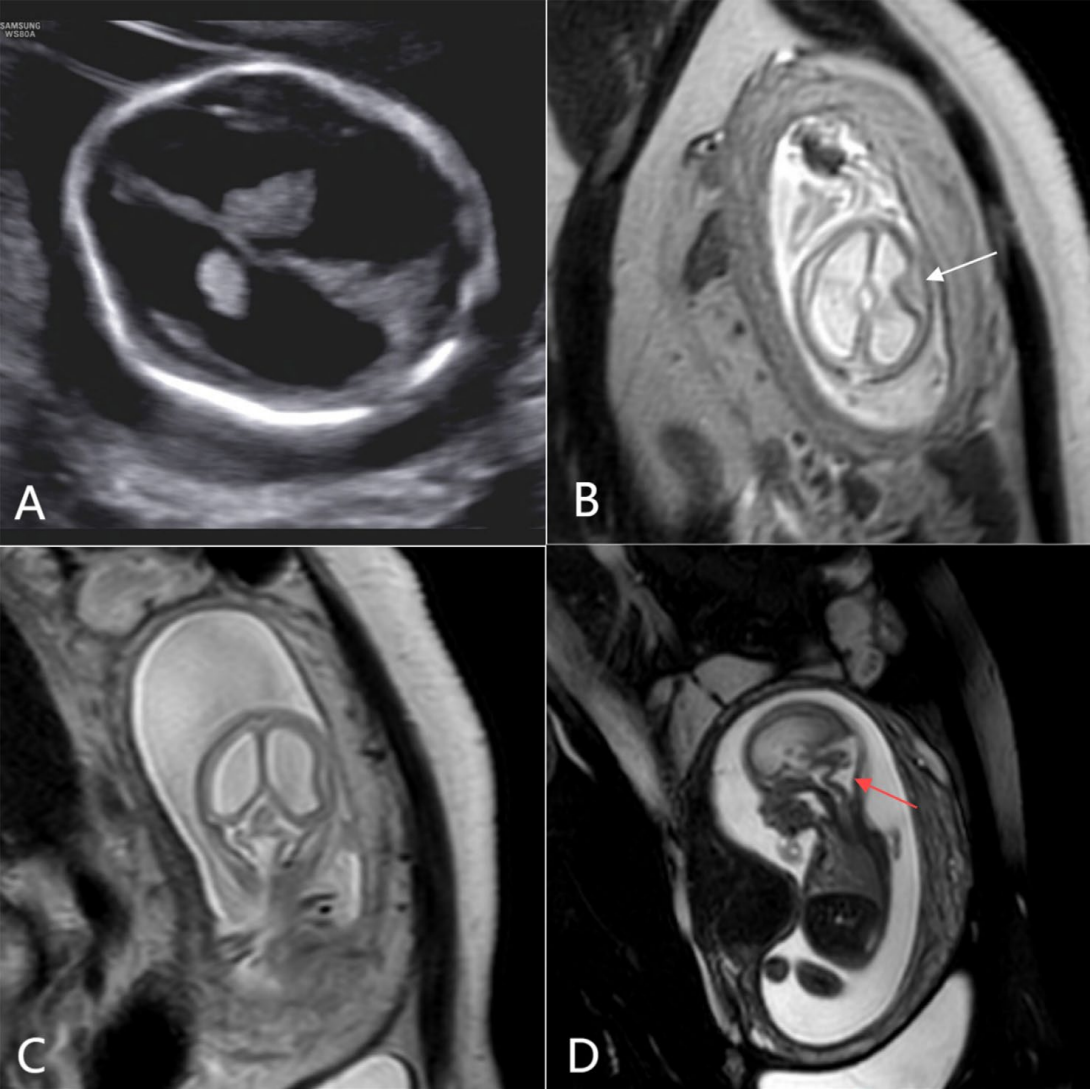
Images from Case 2. (A) US imaging at 17 weeks and 2 days reveals severe hydrocephalus in the lateral ventricle (arrow). (B) In vivo MRI at 17 weeks and 5 days, with axial T2—weighted imaging, demonstrates agyria and the absence of the pericerebral space, resulting in interhemispheric pseudofusion. Severe ventriculomegaly is again associated with periventricular cortical nodular heterotopias, a manifestation of a neuronal migration disorder (arrow). (C) A midline sagittal T2—weighted image shows severe hydrocephalus in the lateral ventricle. (D) The brainstem is hypoplastic and characterized by a dorsal pontomesencephalic kink (red arrow). The third ventricle is dilated, and the corpus callosum is hypoplastic yet intact.

Two years later, the woman became pregnant again. At 13 weeks and 2 days of gestation, US monitoring revealed bilateral choroid plexus asymmetry, a small size, and an abnormal shape of the posterior fossa. At 17 weeks and 2 days of gestation, US revealed hydrocephalus (right and left ventricular sizes: 13 and 11 mm, respectively) and an abnormal cerebellar vermis in the second pregnancy (Figure 2A). At 17 weeks and 5 days of gestation, an MRI was performed using the same 3‐Tesla unit and protocol as employed for the initial fetus. The prenatal MRI revealed several distinctive abnormalities, including a pontomesencephalic kink associated with pontine and cerebellar hypoplasia. Additional findings included hydrocephalus, callosal dysgenesis, cobblestone lissencephaly, and small cerebellar cysts (Figure 2B–D). These observations, coupled with the recurrence of fetal malformations in subsequent pregnancies, strongly indicated the presence of an underlying genetic disorder. Due to the complexity of the case, the pregnancy was terminated at 18 weeks, and whole‐exome sequencing (WES), postmortem MRI, and brain biopsy were recommended.

### 2.2 Prenatal Chromosome Examinations

The presence of structural abnormalities in the fetus warranted genetic testing. Amniocentesis was performed to extract fetal DNA for comprehensive genetic analysis. We employed G‐banding—a conventional cytogenetic technique—along with single nucleotide polymorphism (SNP) arrays, which can identify microdeletions and microduplications, to address the limitations of G‐banding in detecting chromosomal aberrations. The findings indicated a normal fetal karyotype without pathogenic copy number variants (CNVs).

### 2.3 Postmortem MRI and Postmortem Imaging

**FIGURE 3:**
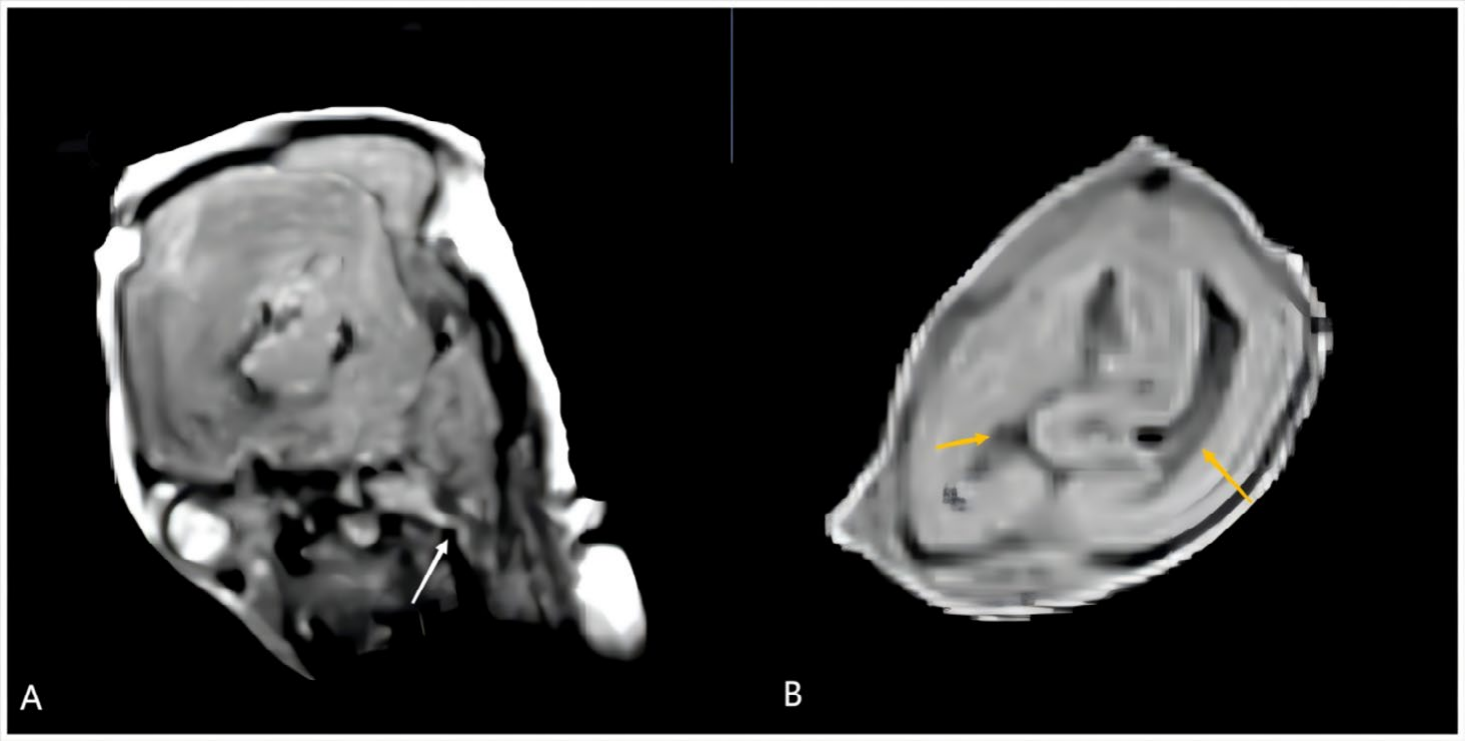
Postmortem MRI findings at 21 weeks and 2 days of gestation. (A) Postmortem MRI reveals cerebellar vermis hypoplasia and a kinked brainstem, indicated by the white arrow. (B) Axial postmortem MRI shows severe hydrocephalus of the lateral ventricles, indicated by the yellow arrows.

MRI revealed an enlargement of the bilateral lateral ventricles and agenesis of the corpus callosum. The cerebellum exhibited bilateral hemispheric hypoplasia and a significantly small vermis, accompanied by a cystic lesion. The brainstem displayed severe dysgenesis, characterized by a primitive ‘Z’‐shaped morphology (Figure 3).

### 2.4 Methods and Results of Brain Autopsy

**FIGURE 4:**
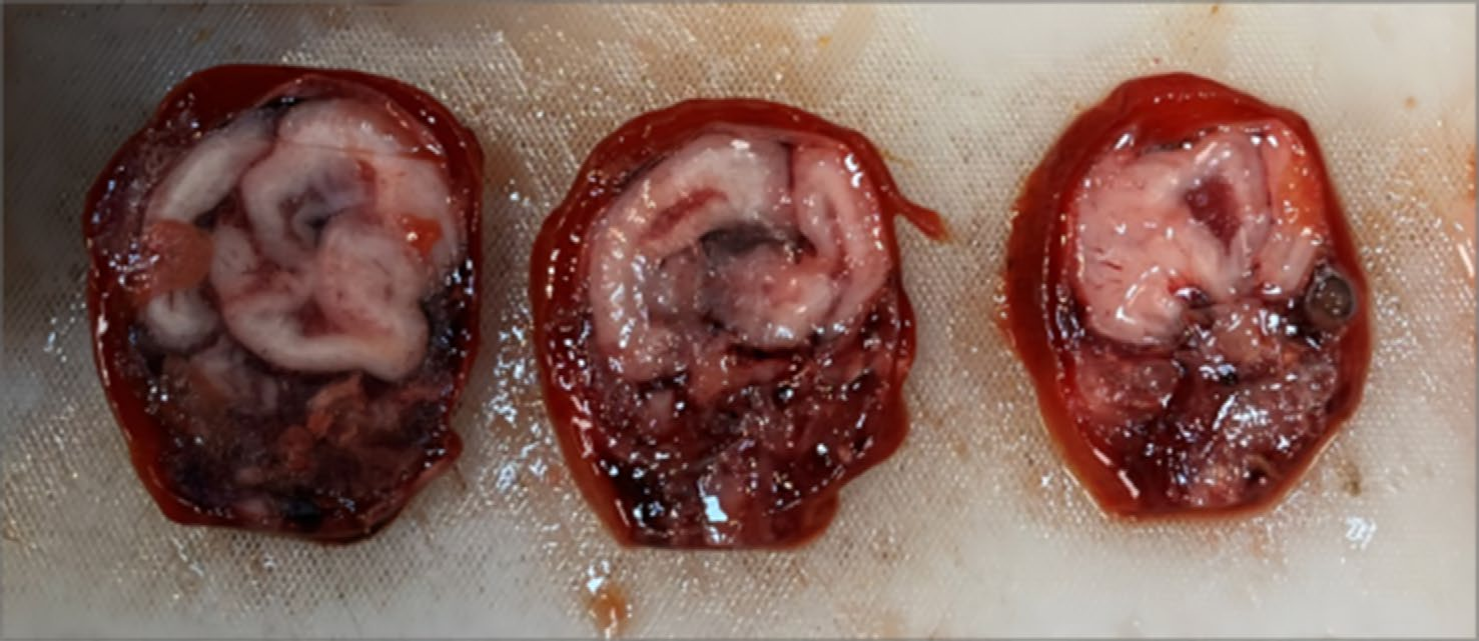
Brain autopsy findings display an agyric and hypoplastic brainstem.

Following the second termination, the fetus was preserved for 1 week in a 10% aqueous phosphate‐buffered formalin solution. Externally, the fetus appeared normal. The brain was subsequently fixed for an additional 10 days post‐extraction. Upon gross examination, the paraffin sections were stained with hematoxylin and eosin. Macroscopically, a 3 mm bone defect was observed in the upper region of the occipital bone, with no evidence of meningocele or encephalocele. Examination of the central nervous system revealed thickened leptomeninges, smooth cortical surfaces, and tri‐ventricular hydrocephalus characterized by the dilatation of the third and lateral ventricles. Additionally, agenesis of the corpus callosum and a kinked brainstem with severe hypoplasia of the cerebellar vermis were observed (Figure 4).

### 2.5 Genomic Analysis by WES‐Trio and RNA Sequencing

**FIGURE 5:**
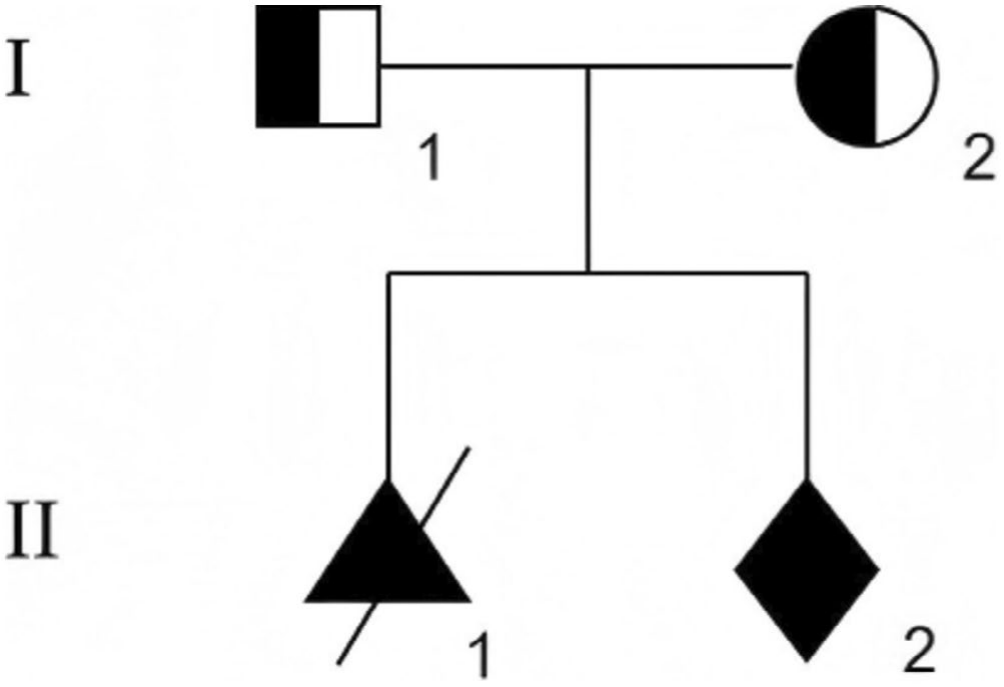
Family pedigree along with the results of Sanger sequencing for the *POMT1*. Both parents have a heterozygous mutation, c.123‐11_123‐5delTTTTTTT, in the *POMT1*, while the fetus (II2) is homozygous for this mutation.

**FIGURE 6:**
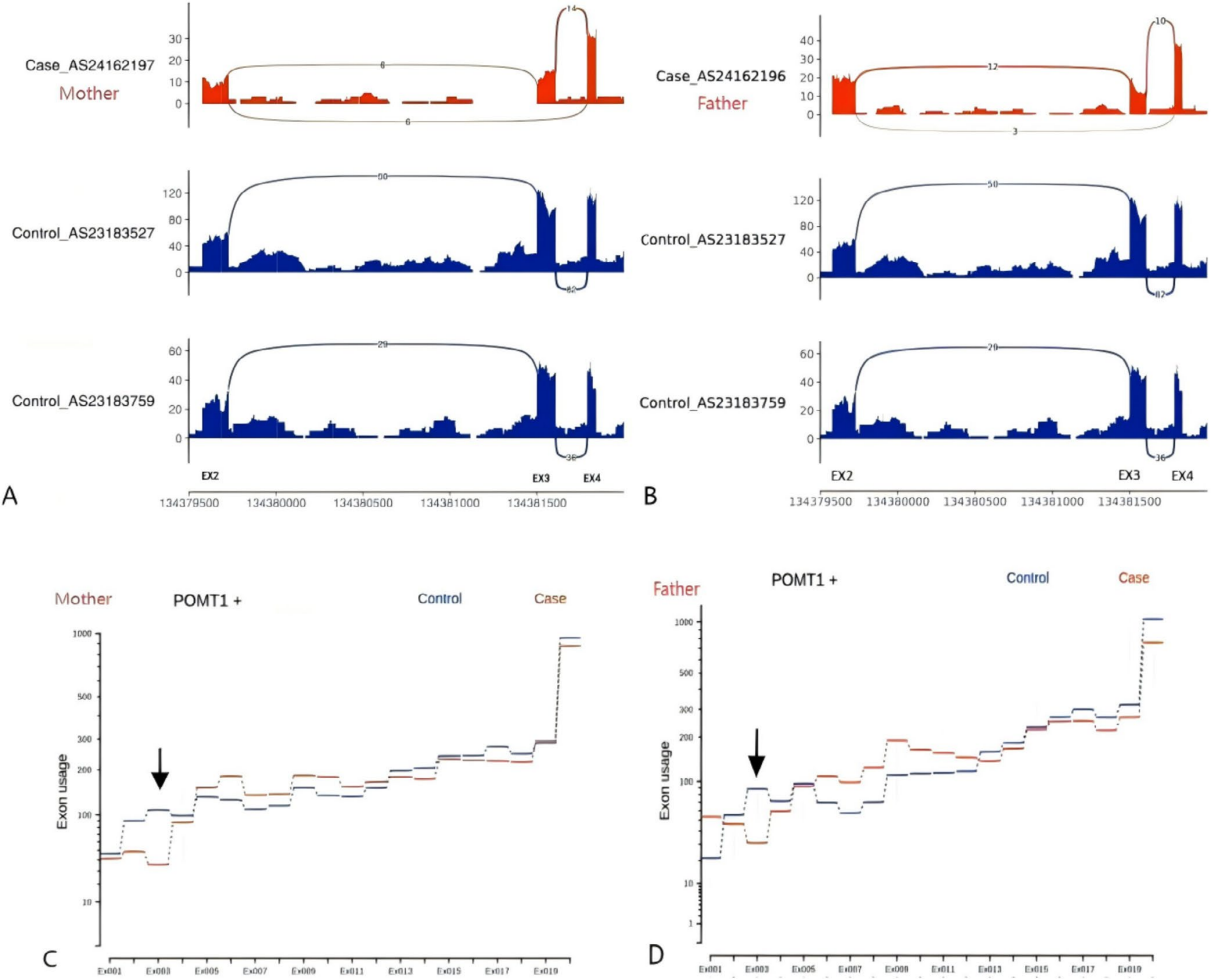
Alternative splicing events occurring in the parents at exon 3 of POMT1 due to the mutation c.123‐11_123‐5del (chr9:134381487, NM_001077365.2) are demonstrated in panels (A, B). RNA—sequencing results show the presence of exon 3 deletion accompanied by exon skipping events. The expression of exon 3 of POMT1 in the parents is significantly lower than that of the control, as shown in panels (C, D).

DNA was extracted from amniotic fluid using the SolPure Blood DNA Kit (Magen) following the manufacturer's instructions. WES was performed using the Illumina HiSeq 2000 platform (Illumina) following the manufacturer's instructions. A homozygous variant, c.123‐11_123‐5del, was identified in POMT1 of the fetus, while both parents were confirmed to be heterozygous for this mutation. These findings were validated using Sanger sequencing (Figure 5). According to the ACMG/AMP criteria, c.123‐11_123‐5del in POMT1 was initially classified as a variant of uncertain significance based on its absence from population databases (PM2) and in silico prediction of splice disruption (PP3; SpliceAI Acceptor Loss: 0.84). To evaluate its effect on splicing, RNA sequencing was performed using available peripheral blood samples from the parents. RNA analysis demonstrated aberrant splicing with reduced exon 3 usage/exon skipping, supporting a loss‐of‐function mechanism. Given that loss of function is an established disease mechanism for POMT1‐related dystroglycanopathy, this RNA evidence supported upgrading the variant classification. Taken together, the variant was reclassified as likely pathogenic according to the applied ACMG/AMP evidence framework (Figure 6).

## 3 Discussion

Mutations in POMT1 can lead to three distinct forms of muscular dystrophy‐dystroglycanopathy (MDDG): a severe congenital form characterized by brain and eye anomalies (type A1; MDDGA1), formerly known as WWS or MEB; a moderately severe congenital form characterized by impaired intellectual development (type B1; MDDGB1); and a milder limb‐girdle form (type C1; MDDGC1). Among these, WWS is the most severe phenotype of congenital autosomal recessive muscular dystrophy. These disorders are characterized by brain malformations, retinal abnormalities, and congenital muscle dystrophies [7, 8]. WWS is associated with fatal outcomes, with most of the affected patients succumbing in infancy [9, 10]. In families with a history of the disorder, the recurrence risk in subsequent pregnancies is 25%. Consequently, early detection and diagnosis of WWS are essential for parents to consider options for prenatal and pre‐implantation genetic diagnosis.

Most previous studies on WWS have concentrated on genetic analysis and US imaging findings. Although ocular anomalies are characteristic of WWS, prenatal diagnosis remains challenging during the second trimester. This is due to the difficulty in assessing characteristic findings, including cobblestone lissencephaly and the kink of the mesencephalic‐pontine junction, through fetal US examination [6]. Conversely, fetal MRI provides superior anatomical depiction of fetal cortical malformations and the posterior fossa [11], facilitating the WWS diagnosis in the early second trimester [4]. To our knowledge, few reports have described combined fetal US and MRI neuroimaging findings of WWS in affected siblings during the early second trimester.

Brain abnormalities in WWS identified through MRI include cobblestone cortex malformations, ventricular dilation or hydrocephalus, abnormal brainstem development (characterized by zigzagging), cerebellar dysplasia, and encephalocele. Our findings align with the existing literature, indicating that type II lissencephaly (cobblestone) is the most prevalent brain anomaly in WWS, characterized by near‐complete agyria. Additionally, midbrain kinking, cerebellar hypoplasia, hydrocephalus, and occipital meningocele are typically observed in WWS cases [12]. Similar brain involvement has been documented in patients with WWS with genetically confirmed dystroglycanopathy caused by mutations in other genes, including *POMT2*, LARGE, POMGnT1, and FUKKUTIN [13, 14]. Notably, gene‐specific patterns of brain malformations in patients with dystroglycanopathies have not been clearly established [15]. Mutations in the same gene can lead to a broad spectrum of clinical and radiological findings, while similar phenotypes may result from mutations in different genes. Although mutations in *POMT1* have been reported in many cases of WWS associated with cleft lip and palate and ocular abnormalities, such mutations were not identified in our case. In this case, despite the pregnant woman's history of adverse pregnancy outcomes and the fetus exhibiting characteristic imaging features of WWS, the limited understanding of the pathogenic genetic mechanisms underlying abnormal brainstem morphology led to an underestimation during US and MRI assessments in the second pregnancy. These findings further support the existence of a similar intrafamilial disease course in families with multiple affected children.

The brainstem exhibits three distinct curvatures at 7 weeks of gestation: the midbrain curvature, the pons curvature, and the cervical curvature, collectively forming an M‐shape. The midbrain and pons curvatures typically regress, resulting in a straight brainstem by approximately 12–13 weeks of gestation. During the second trimester, a ‘Z’‐shaped brainstem is deemed pathological and often suggests significant disruptions in brain development [16, 17, 18]. This condition is more prevalent in WWS and necessitates a comprehensive evaluation of the fetal central nervous system and ocular assessment. In our study, US findings in the early second trimester were nonspecific, primarily detecting ventricular dilation and cerebellar dysplasia. Consistent with previous studies, MRI examinations of both siblings during the second trimester confirmed similar findings, including marked hydrocephalus with an absent septum, occipital cephalocele, ‘Z’‐shaped brainstem, fused colliculi, and hypoplasia of the brainstem and cerebellum, all typically visualized on midline sagittal images. Notably, identifying the absence of complex convolutions on the brain surface as indicative of cobblestone lissencephaly during the second trimester may be challenging, particularly when coexisting hydrocephalus may diminish the sensitivity of cortical sulci detection.

The results of traditional karyotype analysis and SNP array were nonsignificant; however, WES revealed a homozygous deletion variant in POMT1, which was confirmed by RNA sequencing to lead to variable splicing. This ultimately results in the deletion of transcription products from exon 3 and a decrease in expression, potentially causing WWS with associated brain anomalies. Pathogenic missense mutations in POMT1 are the most prevalent and have primarily been reported in pediatric cases. To the best of our knowledge, only two cases have documented the molecular analysis of fetal DNA alongside fetal MRI findings that indicate an abnormally shaped brainstem [6, 19]. R. Achiron demonstrated that early diagnosis of WWS phenotype is feasible as early as 11 weeks of gestation by identifying its characteristic features, confirmed through molecular genetics, post‐abortion MRI, and histopathology [20]. To our knowledge, few reports have integrated antenatal imaging, postmortem MRI, autopsy, and molecular evidence in fetal Walker–Warburg syndrome. The novelty of the present report lies in documenting a recurrent hypoplastic ‘Z’‐shaped brainstem in two affected siblings during the early second trimester and correlating this imaging pattern with a homozygous POMT1 splice‐altering variant. Recognition of this imaging pattern may facilitate earlier diagnostic decision‐making before the full spectrum of typical features becomes apparent. This may be particularly relevant in settings where early pregnancy management decisions are legally and clinically time‐sensitive. Therefore, WWS should be considered when a hypoplastic ‘Z’‐shaped brainstem is identified, particularly in the presence of a positive family history. We also emphasize the importance of preserving critical postmortem samples for genetic testing after pregnancy termination, as this information is crucial for recurrence‐risk counseling and future reproductive planning. This report has several limitations. First, it describes two affected siblings from a single family, which limits the generalizability of the imaging phenotype. Second, genetic testing and autopsy were not available for the first fetus. Third, although RNA sequencing supports aberrant splicing, additional functional studies and independent cases are required to further confirm the pathogenic mechanism and genotype–phenotype correlation.

## 4 Conclusion

This report enhances our understanding of WWS associated with a POMT1 splice‐altering variant by describing a specific fetal imaging pattern observed in two siblings during the second trimester. While advancements in molecular techniques have improved the WWS diagnosis, we emphasize that MRI remains a powerful tool for demonstrating the spectrum of fetal brain malformations. Our case underscores the challenges associated with MRI and histopathologic assessment of WWS during the fetal period. During the second trimester, identifying a ‘Z’‐shaped brainstem should be strongly considered as a potential WWS indicator. Therefore, targeted pathological assessment and genetic screening are essential for accurate diagnosis, recurrence‐risk counseling, and future reproductive decision‐making.

## Author Contributions

**Jing Zhang:** writing – original draft. **Pin Wang:** writing – original draft. **Gan Tian:** supervision. **Yu Hu:** resources. **Xin Zhang:** data curation. **Peng Huang:** funding acquisition. **Xiang Huang:** supervision. **Xiaoqiang Zhou:** software. **Fengying Chen:** data curation.

## Funding

The Guangdong Basic and Applied Basic Research Foundation (2022A1515140127) and the Innovation Project of Women and Children Medical Research Center Affiliated with Foshan Institute of Fetal Medicine (FEYJZX‐2021‐003).

## Ethics Statement

The study protocol received ethical approval from the Medical Ethics Committee of The Affiliated Foshan Women and Children Hospital, Guangdong Medical University.

## Consent

Written informed consent was obtained from the patient's parents/legal guardians for publication of this case report and any accompanying images.

## Conflicts of Interest

The authors declare no conflicts of interest.

## Data Availability

The data that support the findings of this case report are available from the corresponding author upon reasonable request. The data are not publicly available due to privacy or ethical restrictions.

## References

[1] A. A. Abdel Razek , A. Y. Kandell , L. G. Elsorogy , A. Elmongy , and A. A. Basett , “Disorders of Cortical Formation: MR Imaging Features,” American Journal of Neuroradiology 30 (2009): 4–11. 18687750 10.3174/ajnr.A1223 PMC7051699

[2] D. Beltran‐Valero de Bernabe , S. Currier , A. Steinbrecher , et al., “Mutations in the O‐Mannosyltransferase Gene POMT1 Give Rise to the Severe Neuronal Migration Disorder Walker‐Warburg Syndrome,” American Journal of Human Genetics 71 (2002): 1033–1043. 12369018 10.1086/342975 PMC419999

[3] M. Judas , G. Sedmak , M. Rados , et al., “POMT1‐Associated Walker‐Warburg Syndrome: A Disorder of Dendritic Development of Neocortical Neurons,” Neuropediatrics 40 (2009): 6–14. 19639522 10.1055/s-0029-1224099

[4] S. Zago , E. Silvestri , T. Arcangeli , et al., “Fetal Presentation of Walker‐Warburg Syndrome With Compound Heterozygous POMT2 Missense Mutations,” Fetal and Pediatric Pathology 42 (2023): 334–341. 36048137 10.1080/15513815.2022.2116620

[5] A. S. Low , S. L. Lee , A. S. Tan , D. K. Chan , and L. L. Chan , “Difficulties With Prenatal Diagnosis of the Walker‐Warburg Syndrome,” Acta Radiologica 46 (2005): 645–651. 16334849 10.1080/02841850510021409

[6] A. Lacalm , B. Nadaud , M. Massoud , A. Putoux , P. Gaucherand , and L. Guibaud , “Prenatal Diagnosis of Cobblestone Lissencephaly Associated With Walker‐Warburg Syndrome Based on a Specific Sonographic Pattern,” Ultrasound in Obstetrics & Gynecology 47 (2016): 117–122. 26315758 10.1002/uog.15735

[7] M. Warburg , “Heterogeneity of Congenital Retinal Non‐Attachment, Falciform Folds and Retinal Dysplasia. A Guide to Genetic Counselling,” Human Heredity 26 (1976): 137–148. 950240 10.1159/000152795

[8] B. Cormand , H. Pihko , M. Bayes , et al., “Clinical and Genetic Distinction Between Walker‐Warburg Syndrome and Muscle‐Eye‐Brain Disease,” Neurology 56 (2001): 1059–1069. 11320179 10.1212/wnl.56.8.1059

[9] M. C. Manzini , D. Gleason , B. S. Chang , et al., “Ethnically Diverse Causes of Walker‐Warburg Syndrome (WWS): FCMD Mutations Are a More Common Cause of WWS Outside of the Middle East,” Human Mutation 29 (2008): E231‐241. 18752264 10.1002/humu.20844 PMC2577713

[10] R. Shaheen , E. Faqeih , S. Ansari , and F. S. Alkuraya , “A Truncating Mutation in B3GNT1 Causes Severe Walker‐Warburg Syndrome,” Neurogenetics 14 (2013): 243–245. 23877401 10.1007/s10048-013-0367-8

[11] E. M. Simon , R. B. Goldstein , F. V. Coakley , et al., “Fast MR Imaging of Fetal CNS Anomalies In Utero,” AJNR. American Journal of Neuroradiology 21 (2000): 1688–1698. 11039352 PMC8174857

[12] F. Vercellino , I. Meola , M. C. Strozzi , C. Nettuno , C. Lazzotti , and P. Russo , “Brain MR Imaging Findings in Children With Congenital Muscular Dystrophies,” International Journal of Clinical Images and Medical Reviews 1 (2022): 1041.

[13] C. Bouchet , M. Gonzales , S. Vuillaumier‐Barrot , et al., “Molecular Heterogeneity in Fetal Forms of Type II Lissencephaly,” Human Mutation 28 (2007): 1020–1027. 17559086 10.1002/humu.20561

[14] L. Devisme , C. Bouchet , M. Gonzales , et al., “Cobblestone Lissencephaly: Neuropathological Subtypes and Correlations With Genes of Dystroglycanopathies,” Brain 135 (2012): 469–482. 22323514 10.1093/brain/awr357

[15] T. Geis , T. Rodl , H. Topaloglu , et al., “Clinical Long‐Time Course, Novel Mutations and Genotype‐Phenotype Correlation in a Cohort of 27 Families With POMT1‐Related Disorders,” Orphanet Journal of Rare Diseases 14 (2019): 179. 31311558 10.1186/s13023-019-1119-0 PMC6636095

[16] A. Stroustrup Smith , D. Levine , P. D. Barnes , and R. L. Robertson , “Magnetic Resonance Imaging of the Kinked Fetal Brain Stem: A Sign of Severe Dysgenesis,” Journal of Ultrasound in Medicine 24 (2005): 1697–1709. 16301726 10.7863/jum.2005.24.12.1697 PMC1698953

[17] K. K. Haratz and T. Lerman‐Sagie , “Prenatal Diagnosis of Brainstem Anomalies,” European Journal of Paediatric Neurology 22 (2018): 1016–1026. 30448280 10.1016/j.ejpn.2018.06.011

[18] R. Birnbaum , R. Barzilay , M. Brusilov , P. Acharya , G. Malinger , and K. Krajden Haratz , “Early Second‐Trimester Three‐Dimensional Transvaginal Neurosonography of Fetal Midbrain and Hindbrain: Normative Data and Technical Aspects,” Ultrasound in Obstetrics & Gynecology 59 (2022): 317–324. 34002885 10.1002/uog.23691

[19] S. Alharbi , A. Alhashem , F. Alkuraya , F. Kashlan , and K. Tlili‐Graiess , “Neuroimaging Manifestations and Genetic Heterogeneity of Walker‐Warburg Syndrome in Saudi Patients,” Brain & Development 43 (2021): 380–388. 33199158 10.1016/j.braindev.2020.10.012

[20] R. Achiron , E. Katorza , H. Reznik‐Wolf , E. Pras , D. Kidron , and M. Berkenstadtt , “Very Early In‐Utero Diagnosis of Walker‐Warburg Phenotype: The Cutting Edge of Technology,” Ultrasound International Open 2 (2016): E54–E57. 27689171 10.1055/s-0036-1582303 PMC5032857

